# Poor Control of Blood Glucose, Lifestyle, and Cardiometabolic Parameters in Younger Adult Patients with Type 2 Diabetes Mellitus

**DOI:** 10.3390/jcm8091405

**Published:** 2019-09-06

**Authors:** Ga Eun Nam, Byoungduck Han, Chae Lin Joo, Seo Young Kang, Jisun Lim, Yang-Hyun Kim, Hye Soon Park

**Affiliations:** 1 Department of Family Medicine, Korea University Anam Hospital, Korea University College of Medicine, Seoul 02841, Korea (G.E.N.) (Y.-H.K.); 2 Department of Family Medicine, Sahmyook Medical Center, Seoul 02500, Korea; 3 Department of Family Medicine, Asan Medical Center, University of Ulsan College of Medicine, Seoul 05505, Korea (C.L.J.) (S.Y.K.) (J.L.)

**Keywords:** adults, blood glucose, life style, risk factors, type 2 diabetes

## Abstract

This study investigated the awareness, treatment, and control of type 2 diabetes mellitus (T2DM), lifestyle factors, and cardiometabolic parameters according to age groups among patients with T2DM. Data of 1507 patients with T2DM aged ≥35 years in the Korea National Health and Nutrition Examination Survey VI (2013–2015) were analyzed. Multivariable logistic regression models were used to compare the awareness, treatment, and control rate of T2DM and lifestyle and cardiometabolic parameters according to age groups. The adjusted odds ratios (ORs) for lack of awareness about and non-treatment of T2DM, as well as poor glycemic control, were significantly increased with younger age in both men and women. ORs for heavy drinkers and current smokers also significantly increased with younger age in both men and women. The adjusted ORs for high low-density lipoprotein cholesterol (≥100 mg/dL), hypertriglyceridemia (≥150 mg/dL), and obesity significantly increased with younger age in men, but not in women. Among Korean adults with T2DM, awareness, treatment, and control rates of the condition were poorer in younger patients than in older patients. Education regarding the control of glycemia, cardiovascular risk factors, and improvement of lifestyles should be reinforced among younger-aged adults for the long-term management of T2DM.

## 1. Introduction

Type 2 diabetes mellitus (T2DM) is a growing threat to health worldwide. The global prevalence of T2DM in adults has doubled during the past two decades. The number of people with T2DM is anticipated to increase from 415 million in 2015 to 642 million by 2040 [1]. It has been reported that more than 80% of patients with T2DM live in developing countries, and more than 60% live in Asia [2]. In South Korea, 13.7% (4.8 million) of adults aged ≥30 years had T2DM in 2014 [3]. Ageing, urbanization, sedentary lifestyle, and epigenetic changes have contributed to the rapid increase in the burden of T2DM in many developing countries [2].

Controlling T2DM is very important because it is associated with future health outcomes such as microvascular disease, cardiovascular disease (CVD), infection, and sudden death. In a 10-year follow-up study, good glycemic control in patients with T2DM reduced the risk of microvascular disease, cardiovascular complications, and mortality [4]. Lifestyle modification and management of cardiovascular risk factors are essential components to preventing complications in patients with T2DM. The American Diabetes Association guidelines recommend a glycosylated hemoglobin (HbA1c) level of <7% for glycemic control in adults [5]. However, more stringent goals of glycemic control, such as HbA1c <6.5%, may be suggested for patients with short duration of T2DM, long life expectancy, or less severe CVD; these patients are generally younger-aged adults.

Recently, there has been an increase in the prevalence of T2DM among younger people [6]. Despite their shorter duration of diabetes, younger patients with T2DM have a more aggressive disease phenotype, leading to premature development of complications. Mortality in these patients is twice as high as that of age-matched patients with type 1 diabetes mellitus (T1DM) in which CVD is the main cause of death [7]. Young-onset T2DM affects more working age individuals, leading to significant societal effects. In addition, adverse impacts on quality of life and unfavorable long-term outcomes raise the possibility of a future public health catastrophe [6].

There are prominent generation gaps in health behaviors and attitudes about the management of health problems in developing countries owing to rapid changes of environmental factors and socioeconomic growth in a short period. Furthermore, age has an important role in the care of T2DM, in which self-management and compliance contribute to disease control. However, there is limited data about management patterns according to age group among patients with T2DM. Therefore, we investigated the awareness, treatment, and control of T2DM as well as lifestyle and cardiometabolic parameters according to age groups among patients with T2DM, using nationally representative data of the South Korean population.

## 2. Experimental Section

### 2.1. Survey Overview and Study Participants

This population-based cross-sectional study was based on data from the 6th Korea National Health and Nutrition Examination Survey (KNHANES VI) between 2013 and 2015, conducted by the Korea Centers for Disease Control and Prevention. The KNHANES is designed to obtain national health estimates and information about the nutritional status of the South Korean population and consists of three parts: health interviews, health examinations, and nutrition surveys.

Among 22,948 individuals who participated in the KNHANES VI, we initially included 1572 patients with T2DM aged ≥35 years. We excluded individuals with chronic debilitating diseases, such as liver cirrhosis, chronic kidney disease, and cancer (*n* = 65). Finally, 1507 individuals (767 men and 740 women) were included in the analysis. Participants were considered to have T2DM if they were previously diagnosed with T2DM by a physician, or if they were taking oral hypoglycemic agents or insulin, or if their fasting plasma glucose (FPG) level was ≥126 mg/dL [5]. All participants provided written informed consent. The Research Ethics Review Committee of the Korea Centers for Disease Control and Prevention approved the survey protocol (No. 2013-07CON-03-4C, No. 2013-12EXP-03-5C, and No. 2015–01-02-6C).

### 2.2. Assessment and Definitions

Study participants’ lifestyle factors were assessed using a standardized, self-reported questionnaire. We categorized smoking status as current smoker, former smoker, and never smoker. According to the National Institute on Alcohol Abuse and Alcoholism definition, we categorized participants as non-drinker, mild to moderate drinker, and heavy drinker based on their amount and frequency of alcohol consumption [8]. Heavy drinkers were defined as men who consumed >14 glasses/week of an alcoholic beverage and women who consumed >7 glasses/week. Physical activity levels were categorized into high, moderate, or low, according to the number of days and time spent engaged in vigorous-intensity (≥3000 MET-min) and moderate-intensity (600–3000 MET-min) work based on the Korean version of the Global Physical Activity Questionnaire [9]. The nutritional status including daily intakes of total calories, carbohydrates, fat, and protein, was assessed using a 24-h dietary recall questionnaire.

Participants’ height and weight were measured with wearing light clothing, and body mass index (BMI) was calculated as weight (kg) divided by height (m) squared. Waist circumference (WC) was measured at the midline between the bottom of the lowest rib and the upper end of the iliac crest. Blood pressure (BP) was measured three times with a standardized sphygmomanometer after at least 5 min of rest, and the mean of the second and third measurements was calculated. Blood sampling was performed after fasting for at least 8 h. Levels of FPG, total cholesterol (TC), low-density lipoprotein cholesterol (LDL-C), triglycerides, and high-density lipoprotein cholesterol (HDL-C) were measured with a Hitachi 7600 automatic analyzer (Hitachi, Tokyo Japan), and HbA1c was measured using a HLC-723G7 analyzer (Tosoh, Tokyo, Japan).

### 2.3. Awareness, Treatments, and Control of T2DM and Cardiometabolic Parameters

Awareness of having T2DM was considered to be present when participants who were diagnosed with T2DM reported that they had T2DM. Treatment of T2DM was determined as the use of oral hypoglycemic agents or insulin. Uncontrolled T2DM was defined as an HbA1c ≥ 7.0% or FPG ≥ 150 mg/dL. Obesity was defined as BMI ≥ 25 kg/m^2^, according to the Asia–Pacific guidelines for obesity [10]. The definitions of uncontrolled cardiometabolic parameters are as follows: BP ≥ 140/90 mmHg, LDL-C ≥ 100 mg/dL, TG ≥ 150 mg/dL, and HDL-C < 40 mg/dL in men and <50 mg/dL in women [5,11].

### 2.4. Statistical Analyses

Data were extracted from the KNHANES using two-stage stratified cluster sampling. All statistical analyses were carried out using a complex sample model, considering stratification, cluster variables, and weight. We presented the participants’ characteristics according to age groups as mean ± standard error (SE) for continuous variables and number (percentage) for categorical variables. We compared the characteristics according to age groups using one-way analysis of variance (ANOVA) for continuous variables, and the chi-square test for categorical variables. Proportions of unhealthy lifestyle factors, unawareness about and non-treatment of T2DM, poor glycemic control, and uncontrolled cardiometabolic parameters were compared in men and women according to age groups using chi-square tests. The odds ratios (ORs) and 95% confidence intervals (CIs) for unhealthy lifestyle factors, unawareness about and non-treatment of T2DM, poor glycemic control, and uncontrolled cardiometabolic parameters according to age groups were calculated using multivariable logistic regression analyses after adjusting for age, smoking status, alcohol consumption, physical activity, educational level, household income, number of household, and BMI. Statistical analysis was performed using IBM SPSS version 24.0 (IBM Corp., Armonk, NY, USA). *p* < 0.05 was considered statistically significant.

## 3. Results

### 3.1. Characteristics of Study Participants According to Age Group

Table 1 shows the characteristics of the study participants according to age groups. Socioeconomic status was significantly different among age groups in both sexes. The mean body weight and BMI were higher in younger age groups in both men and women. Mean levels of diastolic BP, FPG, HbA1c, TC, and LDL-C tended to be higher in younger adults in both sexes. Daily total calorie intake and the proportions of fat and protein intake increased with younger age for both men and women.

### 3.2. Lifestyle Factors and Obesity According to Age Groups

Table 2 presents the proportions and ORs (95% CIs) of unhealthy lifestyle factors, such as currently smoking, heavy alcohol consumption, and low physical activity, according to age groups. Proportions and adjusted ORs for currently smoking and heavy drinking increased significantly with younger age in both men and women. Proportions and adjusted ORs of low physical activity were not significantly different in men, whereas those in women were lower among younger age groups. Adjusted ORs for obesity were significantly higher in younger men, but not in younger women.

### 3.3. Attitudes and Management of T2DM and Glycemic Control According to Age Groups

**Figure 1:**
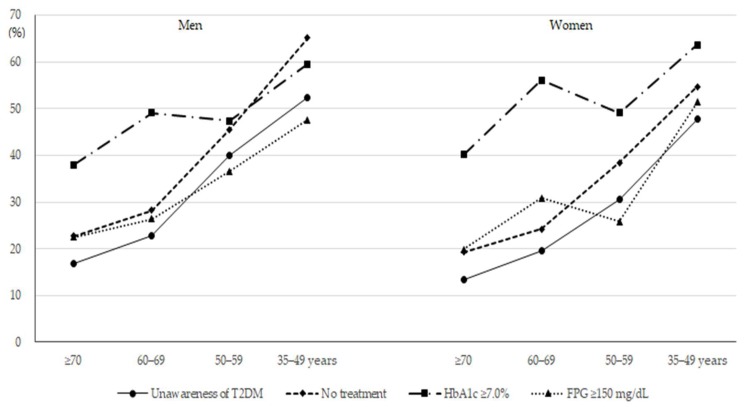
Proportions of unawareness, non-treatment, and poor glycemic control of T2DM according to age groups (all p for trend <0.05).

Awareness, treatment, and control of T2DM according to age groups are shown in Figure 1 and Table 3. The proportions of adults who were unaware of having and were not undergoing treatment for T2DM were significantly higher among younger participants across both sexes. After adjusting for confounding variables, the odds of unawareness and non-treatment of T2DM increased with younger age in both sexes (*p* for trend <0.001). Adjusted ORs for poor glycemic control were 2.45 (95% CI: 1.33–4.48) for HbA1c ≥ 7.0% and 2.95 (1.55–5.61) for FPG ≥ 150 mg/dL among men aged 35–49 years, as compared with those aged ≥70 years. In women, these ORs were 2.31 (95% CI: 1.20–4.47) for HbA1c ≥ 7.0% and 3.98 (2.02–7.84) for FPG ≥ 150 mg/dL. Odds of poor glycemic control tended to be higher among younger age groups in both sexes.

### 3.4. Control of Cardiometabolic Parameters According to Age Groups

Table 4 presents the parameters of cardiometabolic control according to age groups. Adjusted ORs for elevated BP (≥140/90 mmHg) were not significantly different in men whereas, those in women decreased with younger age. Adjusted ORs for elevated LDL-C (≥100 mg/dL) and hypertriglyceridemia (≥150 mg/dL) significantly increased with younger men, but not in younger women. ORs for low HDL-C according to age groups did not differ significantly in both sexes.

## 4. Discussion

This study showed that among patients with T2DM in South Korea, younger adults had poorer attitudes about and management of T2DM as well as poorer glycemic control than older adult patients. Unawareness of their disease among patients with T2DM aged 35–49 years was 4.6–fold higher in men and 3.8–fold higher in women than in their older counterparts aged ≥70 years. Non-treatment of T2DM among patients aged 35–49 years was 6.0–fold higher in men and 3.1–fold higher in women than in their older counterparts. Odds for poor glycemic control were up to 2.5–fold higher in men and 3.98–fold higher in women aged 35–49 years than in their counterparts aged ≥70 years. Furthermore, younger patients with T2DM had more unhealthy lifestyle factors, including current smoking and heavy alcohol consumption than older adult patients. In addition, cardiometabolic parameters, including LDL-C and TG, were found to be more uncontrolled in younger male patients with T2DM.

T2DM in young adults may show an aggressive disease progression, leading to premature complications, despite a younger age and short duration of diabetes [12,13]. Microvascular complications in young patients with T2DM are common at the time of diagnosis and may go through a more aggressive course that in late-onset T2DM [14] or T1DM [7]. In addition, there has been increasing evidence that patients with early-onset T2DM exhibit cardiovascular risk factors and these patients may experience poor cardiovascular outcomes [15,16].

Our results are consistent with previous findings. A recent study among Danish patients with T2DM showed a clear age gradient, with increasing prevalence of clinical risk factors in younger patients with T2DM [17]. In a study among Chinese patients with diabetes, the awareness rate of having T2DM in older patients was higher than that in younger ones [18]. Another study among adults in the United States showed that rates of glycemic control (HbA1c < 7.0%) were better in older patients than in younger patients with T2DM [19]. The higher awareness rates in older patients might be attributed to the availability of greater opportunities to attend health care centers, and more interaction with doctors, as well as being more concerned about their health [20]. On the contrary, younger patients generally do not have enough time to engage in strict T2DM management.

Lifestyle modification is also important in T2DM control. Our study showed that younger patients of both sexes engaged in much more heavy drinking and smoking compared to older patients. These findings might be due to older patients being generally more willing to maintain a healthy lifestyle, to prevent or overcome illness [21]. In terms of physical activity, there was no significant difference according to age groups among men. However, low physical activity was higher in older women than in younger individuals. This finding is likely related to the higher prevalence of musculoskeletal comorbidities in older women. In our study, the prevalence of obesity was higher in younger patients compared with older patients in men, but not in women. This may be because body weight and abdominal circumference in older women tend to increase abruptly after menopause.

Control of cardiovascular risk factors is an important goal in patients with diabetes to prevent micro- and macrovascular complications. Early-onset T2DM is associated with a higher prevalence of retinopathy [22] and higher risk conditions of premature CVD compared to late-onset T2DM [16]. Although the absolute risk for CVD is higher in older adults than in younger ones, younger patients with early-onset T2DM have a higher relative risk of CVD than their age-matched controls. Our study showed that management of LDL-C and TG was poorer in younger than in older men. In women, significant differences in the control of cardiovascular risk factors were not observed among the age groups, except for blood pressure, which was more poorly controlled in older adults than younger ones. These findings are consistent with sex differences found in ORs according to age groups for obesity and the relative higher prevalence of heavy drinking and current smoking among men than women.

Our findings that younger patients with T2DM had unfavorable attitudes towards the management of T2DM and lifestyles, and metabolic parameters related to the condition, suggests that careful monitoring and appropriate management for all these traits are warranted, especially for this age group of patients in clinical practice. Our results indicate that policies to increase the awareness, treatment, and control rates of T2DM among younger adults are urgently required. Strict glycemic control is important for younger patients to reduce complications at an individual level, as well as to help lower future medical costs at the societal level. It is essential to educate young patients with T2DM about healthy lifestyles and to establish a health system that helps these patients maintain healthy behaviors. Initiation of treatment and interventions to maintain a healthy lifestyle will ultimately increase the control rates of T2DM [23]. A structured occupational therapy intervention may be helpful in improving clinical and psychosocial outcomes among individuals with diabetes [24]. Prospective studies investigating the factors affecting the control of T2DM among younger patients with diabetes will help to improve the quality of management in these patients.

There are several limitations in this study. First, as this was a cross-sectional study, we could not establish any cause-and-effect relationships. Second, we could not thoroughly exclude the effects of confounding variables. For example, it is likely that older patients are prescribed multiple medications by health care providers, whereas lifestyle modification rather than hypoglycemic agents is more frequently recommended in younger patients, even those with high glucose levels or cardiovascular risk factors. Third, we did not collect data on the onset time of T2DM because the original national survey did not include it. Therefore, we could not precisely differentiate early- or late-onset T2DM among the study participants. Although onset time was unclear among our older patients, the early-onset of disease in our younger patients (age 35–49 years) was relatively certain. Lastly, we could not exclude recall bias in the diet and lifestyle survey. Despite these limitations, our study includes a nationally representative sample of South Koreans and suggests epidemiologic implications regarding many characteristics of younger adult patients with T2DM. The relatively large sample in this study enabled us to adjust several confounding variables, and to conduct our analysis according to sex and age groups.

## 5. Conclusions

In conclusion, younger adult patients with T2DM had lower awareness about, underwent less treatment for, and experienced poor control rates of T2DM; they also had more unhealthy lifestyles. Young male patients with T2DM showed poorer control of body weight and lipid profiles, compared to older men. Appropriate interventions are urgently needed for younger adult patients with T2DM in South Korea.

## Figures and Tables

**Table 1:** Cardiometabolic and nutritional characteristics according to age groups among patients with T2DM.

	Men (*n* = 767)					Women (*n* = 740)				
	≥70 (*n* = 200)	60–69 (*n* = 282)	50–59 (*n* = 171)	35–49 (*n* = 114)	*p* for Trend	≥70 (*n* = 271)	60–69 (*n* = 238)	50–59 (*n* = 135)	35–49 (*n* = 96)	*p* for Trend
Socioeconomic status										
Education >12 years	32 (18.6)	46 (16.6)	43 (24.4)	54 (43.3)	<0.001	7 (2.6)	8 (3.1)	15 (9.9)	33 (34.3)	<0.001
Income (the highest quartile)	18 (11.1)	59 (20.5)	57 (33.4)	32 (26.6)	0.001	25 (8.9)	28 (11.3)	35 (23.8)	28 (32.2)	<0.001
Single household	16 (6.6)	28 (8.4)	17 (8.5)	8 (8.3)	0.938	100 (32.9)	36 (12.5)	8 (5.7)	2 (2.5)	<0.001
Anthropometric measurements										
Height (cm)	165.2 ± 0.4	166.7 ± 0.4	168.6 ± 0.5	172.8 ± 0.7	<0.001	151.3 ± 0.4	153.7 ± 0.4	155.0 ± 0.6	157.8 ± 0.6	<0.001
Weight (kg)	66.2 ± 0.7	68.5 ± 0.6	71.9 ± 0.8	78.5 ± 1.6	<0.001	57.9 ± 0.6	60.3 ± 0.6	61.5 ± 1.0	65.9 ± 1.5	<0.001
BMI (kg/m^2^)	24.2 ± 0.2	24.6 ± 0.2	25.3 ± 0.3	26.2 ± 0.5	<0.001	25.3 ± 0.2	25.5 ± 0.2	25.6 ± 0.4	26.4 ± 0.5	0.040 *
WC (cm)	88.1 ± 0.7	88.1 ± 0.6	88.9 ± 0.7	90.4 ± 1.1	0.030 *	87.0 ± 0.7	85.8 ± 0.6	84.0 ± 0.9	85.5 ± 1.2	0.108
Cardiometabolic parameters										
Systolic BP (mmHg)	127.1 ± 1.4	124.3 ± 1.1	125.8 ± 1.33	123.2 ± 1.4	0.144	129.4 ± 1.1	129.7 ± 1.4	121.5 ± 1.3	114.7 ± 1.2	<0.001
Diastolic BP (mmHg)	69.2 ± 0.7	74.3 ± 0.7	81.8 ± 0.86	84.2 ± 0.8	<0.001	68.5 ± 0.9	74.3 ± 0.7	76.1 ± 0.8	76.9 ± 1.0	<0.001
FPG (mg/dL)	132.3 ± 2.4	140.1 ± 2.1	147.9 ± 3.1	162.9 ± 4.4	<0.001	130.9 ± 2.5	142.7 ± 3.1	141.3 ± 3.7	162.7 ± 5.3	<0.001
HbA1c (%)	7.04 ± 0.10	7.31 ± 0.08	7.42 ± 0.12	7.87 ± 0.20	<0.001	7.10 ± 0.07	7.50 ± 0.11	7.25 ± 0.12	7.78 ± 0.18	0.002
TC (mg/dL)	171.0 ± 2.4	177.1 ± 2.4	181.2 ± 3.63	206.2 ± 5.0	<0.001	179.3 ± 2.5	182.9 ± 3.6	197.2 ± 4.0	192.6 ± 4.9	<0.001
TG (mg/dL)	152.1 ±10.2	185.2 ± 12.6	226.8 ± 12.9	290.7 ± 23.8	<0.001	143.7 ± 4.7	168.7 ± 8.5	175.1 ± 10.9	148.6 ± 9.0	0.222
LDL-C (mg/dL)	98.9 ± 3.3	104.0 ± 2.8	103.7 ± 3.50	119.0 ± 5.5	0.003	107.2 ± 3.5	108.7 ± 4.6	116.4 ± 4.4	119.0 ± 5.5	0.046
HDL-C (mg/dL)	43.6 ± 0.8	44.3 ± 0.7	43.1 ± 0.70	44.1 ± 1.0	0.954	46.4 ± 0.8	46.8 ± 0.8	47.7 ± 0.9	48.3 ± 1.3	0.160
Daily dietary intake										
Total calorie (kcal)	1824.3 ± 54.4	2080.1 ± 53.9	2301.2 ± 88.9	2389.4 ± 109.2	<0.001	1394.3 ± 49.5	1574.5 ± 49.0	1755.8 ± 70.9	1735.2 ± 77.1	<0.001
Carbohydrate (%)	69.4 ± 1.1	65.0 ± 0.9	61.5 ± 1.3	60.3 ± 1.5	<0.001	74.2 ± 0.8	72.9 ± 1.0	67.3 ± 1.2	63.5 ± 1.6	<0.001
Fat (%)	13.2 ± 0.7	14.8 ± 0.6	15.5 ± 0.7	18.4 ± 1.0	<0.001	12.4 ± 0.6	13.2 ± 0.6	17.3 ± 0.9	19.8 ± 1.0	<0.001
Protein (%)	13.3 ± 0.5	13.1 ± 0.3	13.7 ± 0.4	14.3 ± 0.4	0.018 *	12.3 ± 0.3	12.9 ± 0.3	14.6 ± 0.5	14.4 ± 0.5	<0.001

Abbreviations: T2DM, type 2 diabetes mellitus; BMI, body mass index; WC, waist circumference; BP, blood pressure; FPG, fasting plasma glucose; HbA1c, glycated hemoglobin; TC, total cholesterol; TG, triglyceride; LDL-C, low-density lipoprotein cholesterol; HDL-C, high-density lipoprotein cholesterol. Values are presented as mean ± standard error (SE) or percentage (SE). * *p* values were calculated using post-hoc analysis.

**Table 2:** Lifestyle factors and obesity according to age group among patients with T2DM.

		≥70 Years	60–69 Years	50–59 Years	35–49 Years	*p* for Trend
**Men**						
Current smoker	*n* (%)	37 (19.1)	95 (36.5)	80 (49.1)	62 (57.8)	<0.001
	OR (95% CI)	1 (ref.)	2.63 (1.50–4.61)	4.57 (2.46–8.50)	6.94 (3.44–14.00)	<0.001
Heavy drinker	*n* (%)	21 (11.4)	54 (19.9)	54 (30.2)	37 (36.3)	<0.001
	OR (95% CI)	1 (ref.)	2.03 (1.20–3.42)	1.90 (1.06–3.40)	2.76 (1.38–5.49)	0.010
Low physical activity	*n* (%)	152 (75.4)	189 (68.0)	130 (77.3)	78 (68.6)	0.931
	OR (95% CI)	1 (ref.)	0.73 (0.45–1.18)	1.25 (0.70–2.22)	0.91 (0.48–1.74)	0.640
BMI ≥ 25 kg/m^2^	*n* (%)	70 (35.2)	125 (45.3)	91 (51.6)	67 (57.0)	<0.001
	OR (95% CI)	1 (ref.)	1.57 (1.01–2.45)	2.17 (1.32–3.57)	2.80 (1.60–4.90)	<0.001
**Women**						
Current smoker	*n* (%)	10 (4.0)	15 (6.2)	8 (6.5)	11 (9.6)	<0.001
	OR (95% CI)	1 (ref.)	2.66 (0.81–8.71)	4.06 (1.03–16.00)	7.60 (1.71–33.88)	0.005
Heavy drinker	*n* (%)	5 (2.6)	4 (1.3)	9 (8.1)	10 (10.8)	<0.001
	OR (95% CI)	1 (ref.)	2.44 (1.55–3.85)	3.68 (1.99–6.81)	6.41 (3.22–12.74)	<0.001
Low physical activity	*n* (%)	237 (89.2)	186 (77.5)	111 (83.9)	69 (68.7)	<0.001
	OR (95% CI)	1 (ref.)	0.46 (0.26–0.84)	0.69 (0.35–1.38)	0.31 (0.14–0.70)	0.016
BMI ≥ 25 kg/m^2^	*n* (%)	128 (47.6)	119 (50.8)	66 (47.9)	54 (55.2)	0.368
	OR (95% CI)	1 (ref.)	1.21 (0.77–1.89)	0.98 (0.58–1.66)	1.13 (0.59–2.17)	0.872

Abbreviations: T2DM, type 2 diabetes mellitus; OR, odds ratio; CI, confidence interval; body mass index, BMI. Number (%) are presented as unweight number and weighted percentage. ORs (95% CIs) were calculated after adjusting for education, income, and number of household.

**Table 3:** ORs (95% CIs) of unawareness, non-treatment, and poor glycemic control of T2DM according to age groups.

	≥70 Years	60–69 Years	50–59 Years	35–49 Years	*p* for Trend
**Men**					
Unawareness of T2DM	1 (ref.)	1.34 (0.80–2.24)	2.88 (1.56–5.34)	4.63 (2.45–8.74)	<0.001
Non-treatment	1 (ref.)	1.29 (0.81–2.07)	2.67 (1.50–4.74)	5.99 (3.18–11.30)	<0.001
Poor glycemic control					
HbA1c ≥7.0%	1 (ref.)	1.54 (0.98–2.41)	1.43 (0.84–2.46)	2.45 (1.33–4.48)	0.014
FPG ≥150 mg/dL	1 (ref.)	1.19 (0.70–2.02)	2.02 (1.14–3.59)	2.95 (1.55–5.61)	<0.001
**Women**					
Unawareness of T2DM	1 (ref.)	1.42 (0.81–2.50)	2.25 (1.16–4.39)	3.76 (1.84–7.68)	<0.001
Non-treatment	1 (ref.)	1.29 (0.77–2.18)	2.17 (1.22–3.87)	3.14 (1.59–6.19)	<0.001
Poor glycemic control					
HbA1c ≥7.0%	1 (ref.)	1.51 (0.99–2.31)	1.14 (0.69–1.90)	2.31 (1.20–4.47)	0.048
FPG ≥150 mg/dL	1 (ref.)	1.71 (1.06–2.76)	1.26 (0.71–2.24)	3.98 (2.02–7.84)	0.001

Abbreviations: T2DM, type 2 diabetes mellitus; OR, odds ratio; CI, confidence interval; HbA1c, glycated hemoglobin; FPG, fasting plasma glucose. The number (%) are presented as unweight number and weighted percentage. ORs (95% CIs) were calculated after adjusting for physical activity, alcohol consumption, smoking status, education, income, number of household, and body mass index.

**Table 4:** Control of cardiometabolic parameters according to age groups among patients with T2DM.

		≥70 Years	60–69 Years	50–59 Years	35–49 Years	*p* for Trend
**Men**						
BP ≥140/90 mmHg	*n* (%)	50 (24.5)	53 (18.1)	52 (31.7)	37 (30.6)	0.044
	OR (95% CI)	1 (ref.)	0.59 (0.35–1.01)	1.33 (0.70–2.50)	1.28 (0.64–2.55)	0.091
LDL-C ≥100 mg/dL	*n* (%)	94 (47.2)	145 (52.9)	86 (48.3)	80 (70.5)	0.001
	OR (95% CI)	1 (ref.)	1.59 (0.86–2.92)	1.53 (0.76–3.10)	3.09 (1.54–6.23)	0.005
TG ≥150 mg/dL	*n* (%)	77 (37.2)	129 (49.0)	103 (62.5)	84 (75.8)	<0.001
	OR (95% CI)	1 (ref.)	1.31 (0.86–2.01)	2.24 (1.25–4.02)	4.37 (2.32–8.23)	<0.001
HDL-C <40 mg/dL	*n* (%)	82 (41.6)	101 (37.6)	67 (39.4)	42 (35.8)	0.493
	OR (95% CI)	1 (ref.)	0.83 (0.53–1.31)	0.87 (0.50–1.52)	0.76 (0.42–1.40)	0.489
**Women**						
BP ≥140/90 mmHg	*n* (%)	69 (25.8)	65 (25.7)	23 (14.0)	8 (9.9)	<0.001
	OR (95% CI)	1 (ref.)	1.02 (0.62–1.65)	0.48 (0.25–0.92)	0.28 (0.11–0.72)	0.001
LDL-C ≥100 mg/dL	*n* (%)	137 (50.4)	120 (50.2)	84 (63.3)	69 (70.3)	0.001
	OR (95% CI)	1 (ref.)	0.98 (0.48–1.98)	1.85 (0.87–3.97)	1.50 (0.57–3.98)	0.179
TG ≥150 mg/dL	*n* (%)	100 (34.1)	114 (46.6)	62 (48.9)	40 (43.1)	0.088
	OR (95% CI)	1 (ref.)	1.91 (1.22–2.99)	2.12 (1.17–3.82)	1.35 (0.69–2.64)	0.149
HDL-C <50 mg/dL	*n* (%)	188 (67.5)	153 (66.5)	90 (67.7)	60 (60.8)	0.398
	OR (95% CI)	1 (ref.)	1.11 (0.66–1.87)	1.56 (0.88–2.76)	1.18 (0.60–2.32)	0.328

Abbreviations: BP, blood pressure; LDL-C, low density lipoprotein cholesterol; TG, triglyceride; HDL-C, high density lipoprotein cholesterol; BMI, body mass index. Number (%) are presented as unweight number and weighted percentage. ORs (95% CIs) were calculated after adjusting for physical activity, alcohol consumption, smoking status, education, income, number of household, and body mass index.
